# A Simple Model of Ostracism Formation

**DOI:** 10.1371/journal.pone.0094333

**Published:** 2014-04-29

**Authors:** Pilwon Kim

**Affiliations:** Ulsan National Institute of Science and Technology (UNIST), Department of Mathematical Sciences and School of Technology Management, Ulsan Metropolitan City, South Korea; University of Debrecen, Hungary

## Abstract

We study formation of ostracism in a society from a game theoretical perspective. The dynamics of group formation is complicated in that the choices of the individuals and the form of the groups mutually affect each other in the process. A suggested simple model shows that individual efforts to increase his/her own sense of belonging is responsible for both growth of groups and creation of an outcast. Once a person happens to get behind in synchronizing with others, tendency to alienate him may grow among others, possibly making him left out in the end. Alienating minority occurs even when there is a penalty for disliking and people are encouraged to favor others. Considering that the target is accidentally picked, we can understand ostracism as an inherent part of the group formation, rather than a result of specific discrepancy among people. Another finding is that a single individual who seeks for unconditional unification of the society (“philanthropist”) likely invites his/her own isolation from the society, while the existence of such person generally promotes coalition of others.

## Introduction

Alienation of an outcast is a pervasive and persistent phenomenon in social sphere of life. Ostracism, or social rejection, have devastating results on both individual and social welfare in the long run. For example, neurological research shows that social exclusion activates responses analogous to pain responses associated with physical injury [1], [2].

Ostracised minorities throughout history have included almost every imaginable group of people differing in genders, religions, races, nations, or political beliefs. However, in a daily life, outcasting is often triggered by trifling matters and a victim may be picked without a particular reason [3]. This suggests that creation of ostracism reflects an inherent aspect of group formation.

**Figure 1:**
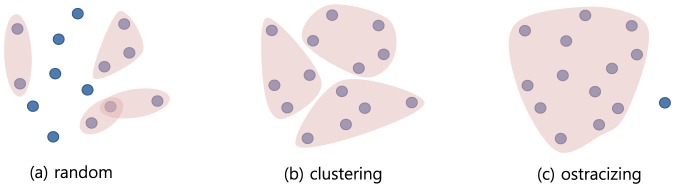
Typical group formations.

Group formation is one of the important economic problems such as resource management, cartel coalition and political lobbying [4]. In various situations, individuals organize themselves in communities to maximize their utilities. Figure 1 illustrates some typical group formations: starting from arbitrary configuration (a), people may organize a couple of similar-sized groups like (b), or sometimes merge into a large group, ostracising one like (c).

There is an extensive body of modeling work on the group formations in the context of both the game theory and the network theory, [5], [6], [7], [8], [9], [10], [11], [12], to name a few. In most researches related to ostracism, it is treated as a powerful tool for mitigating free-riding [13], [14]. Social exclusion is an effective means of social punishment in that ostracised individuals cannot reap the benefits of group efforts.

This article focuses on creation of ostracism as a part of group formation, and develops a simple dynamic coalition model to trace it. We argue that both group formation and outcast occurrence are originated from individual efforts to increase his/her own sense of belonging. Among the most powerful human motives is the desire to form and maintain social bonds [15]. It is argued that the need for belongingness is the need for not only maintaining social contacts but also sharing preferences and beliefs. This is consistent with the cooperative game theory literature [16], [17], [18] which has analysed the trade-off between economies of scale and the cost of heterogeneity in large groups.

We formulate the sense of belonging of a person as 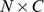, where 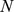 denotes the size of the group that the person belongs to and 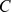 is the congruity that the person experiences in the corresponding group. Individuals decide to like or dislike others, which naturally determines the group that he/she belongs to. The congruity is basically defined as the average distance of the person's preference from those of the others' in the group. People adjust their preferences in repeated games, keeping balance between increase of the group size and increase of the congruity for maximum sense of belonging.

It is obvious that one of the Nash equilibria of the game is the configuration that everyone is synchronized in liking everyone else and therefore belongs to the one same group. However, while people are organizing larger and larger groups, sometimes one or two persons happen to get behind. Then people may start to synchronize their negative attitudes toward the late-joiners. Once established, this tendency is only accelerated along group formation, creating ostracism. It is a robust phenomenon that frequently occurs even when there is a penalty for disliking.

In this work, we also study influence of philanthropy on group formation. Historical review shows that philanthropy has been the primary resource that frees civil society from purely market-driven behavior [19]. We define a philanthropist as one who pursues unconditional bonds with others, no matter how they differ from him/her. In contrast to people who conservatively and selectively adjust their social connections to keep congruity of their groups, a philanthropist is only interested in affecting as many people as possible and therefore contributes to improvement of overall social integration. However, it turned out that philanthropists unavoidably expose themselves to a risk of being an outcast from the society.

## Methods

Imagine there is a set of 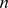 individuals 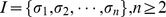. Each individual is characterized by his/her feeling toward other people. For simplicity we assume that there are only two types of feeling toward each individual: friendliness and hostility. We use a “feeling vector” 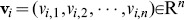 to express the feeling of an individual 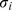 toward people, where 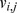 represents an attitude of 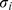 to 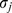. The value of 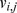 is assigned as
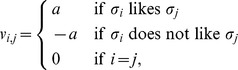
with some 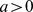.

For each 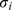, we naturally define the group 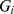 as
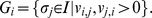
This means that 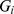 is the set of people who are in mutual favor with 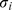. We further set a group 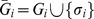 including him/herself.

People feel more intimacy when they share what they like and what they do not with the other group members. That is, in our setup, if they like/dislike the same people together, they feel that their group is more integrated. This observation suggests that congruity 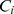 that the individual 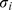 experiences in the group 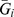 can be defined as an overall similarity between 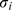 and the members of 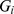. We set
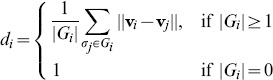
an average distance between the feeling vector of 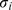 and those of the group members, where 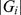 is the number of the individuals in 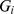. Then the congruity of 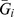 is defined as 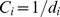. Note that, if we use 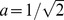, the range of 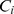 is
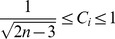
where the lower and upper bounds are attained when the feeling vector of 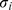 is completely synchronized and desynchronized, respectively, with those of other members in 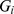.

Now we are ready to define a measure of a sense of belonging. People tend to feel safe and comfortable when staying with similar people. This implies that homogeneity of the group is a factor that positively affects a sense of belonging. It is also natural that, under the same level of homogeneity, the emotion is stronger when the group size is larger. These observations enable us to define a sense of belonging that 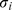 has in 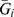 as
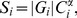
(1)for some constant parameter 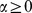.

Note that the feeling vectors 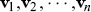 completely characterizes the current configuration and determines the group distribution, 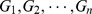, and sense of belonging 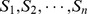. Our basic assumption on group formation is that each individual keeps adjusting emotional attitudes toward others to maximize his/her sense of belonging. To trace dynamics of group formation, we use the best response rule: individuals, when they get a revision chance, adopt their best possible attitude toward others (best response) to the current configuration. This implies that an individual 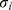 updates the feeling vector 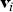 to, say 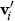, so that the virtual configuration 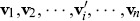 leads to maximization of 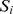. For practical consideration, we assume that one changes his/her attitude toward only one person at a time. That is, for each 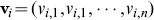, one picks just one component and switch its value from 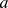 to 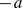 (or the other way around). If there are more than two possible choices for maximum, one of them is arbitrarily selected.

The dynamical behaviour in evolutionary games generally depends on the choice of the update rules as well. In this work, we apply the synchronous update rule: in discrete time steps, the whole individuals 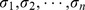 adjust their feeling vectors 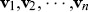 simultaneously according to the best response rule mentioned above. However, it turned out that basic properties of the system are not influenced by update rules. The simulation results that follow in the next sections are qualitatively same for the sequential/random update rules.

## Results and Discussion

### Exclusion of an Accidental Outcast

**Figure 2:**
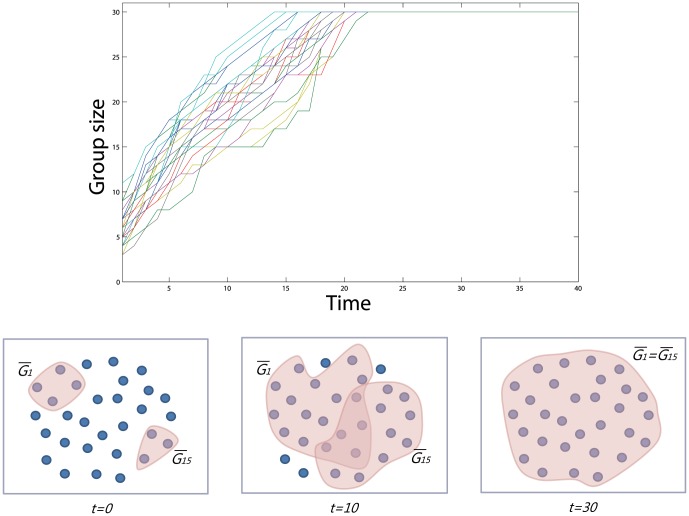
Evolution of the group sizes (upper) and the group formation. Two exemplary groups are illustrated below. The initial ratio of friendliness is 0.4 and the parameter 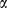 = 1 is used.

In this section, we show that individual efforts to increase their sense of belonging lead to formation of groups, and moreover, to frequent occurrence of ostracism. People's preference to large and homogeneous groups is well explained from the form of the payoff in (1). In Figure 2, one can see a typical group formation of 30 people with the payoff functions and the update rule described in the previous section. The graphs in the upper figure are the evolving group sizes 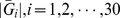 along this discrete time steps upto 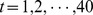. In the initial configuration at 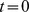, we set the initial ratio of friendliness as low as 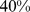 which implies that the probability of mutual friendliness between two arbitrary persons is 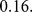 This explains that the most group sizes are around 5 at 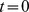. However, initiated from an arbitrary configuration, people start to gather according to the evolutionary rule described in Section 2 and soon melt into a large group before 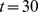. The lower three figures show snapshots of two groups 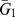 and 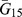 at 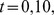 and 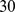, respectively.

**Figure 3:**
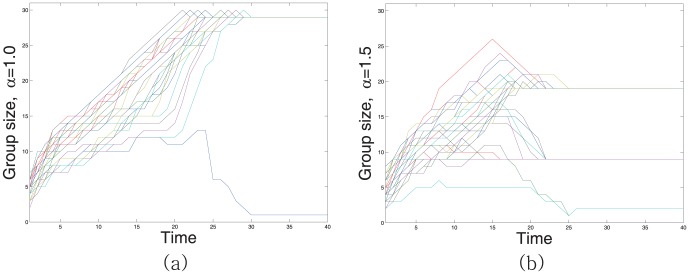
Group division according to the parameter 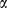. The initial ratio of liking among people is 1/3. When 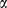 = 1 as in (a), 30 People join together likely making an outcast. In (b), 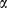 = 1.5 and people break into three groups of 19, 9 and 2.

One of the important observations in this work is that the individual efforts to raise the sense of belonging frequently cause exclusion of others. Suppose that a majority of a group 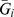, including 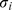, happen to be unfriendly to a certain outsider, say 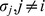 at a certain time. There are generally two possible choices for 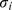 to increase his/her sense of belonging. Firstly, he/she can try turning the attitude toward 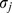 positively to have 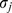 join 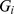. This may increase the group size, while it takes a risk of lowering the congruity 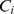. The second choice is that he may keep hating 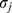, in the expectation that other people in 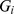 cooperate in refusing 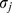. This may keep or even raise the congruity level. Once the second tendency is established, it is only accelerated by more and more people gathering together, eventually making 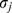 an outcast. Figure 3(a) illustrates typical occurrence of ostracism.

It is notable that the parameter 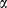 in (1) reflects people's general attitude toward group formation. Note that an individual 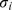 update 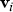 according to the gradient of 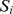. Suppose 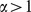. Since the congruity 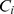 does not exceed 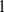, 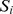 increases rapidly as 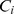 is close to 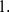 Considering the definition of the sense of belonging, this roughly implies that people can raise their sense of belonging more quickly by synchronizing themselves with those who are already similar to them. In other words, people prefer to be identical with others even if their group is not a large one. This naturally results in a couple of non-overlapping clusterings or exclusive partitions. You can refer to Figure 3(b).

If 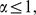 on the contrary, the 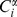 more sensitively reacts to the change in the distant relations. Roughly speaking, people generally mind if they are too far away from others. This tendency dramatically improves social integrity and make emergence of larger groups possible. Unfortunately, it still cannot overcome possible occurrence of ostracism as in Figure 3(a). Especially when the initial configuration of relations are badly biased to hostility, there is a high chance of accidental creation of outcasts.

**Figure 4:**
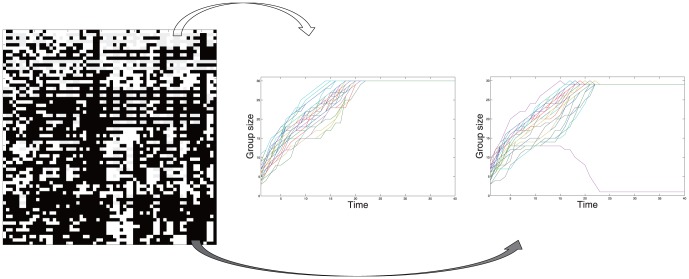
A subset of the initial configuration spaces (left) and two examples of the group formation. The results from 64^2^ different initial configurations are visualized in the checker board. The white cell represents an initial configuration that leads to one entire group, while the black represents one that ends up forming ostracism. The ratio of liking among people at *t* = 0 is 1/3 and the parameter 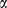 = 1 is used.

Note that the described group formation is based on synchronized best response rule and therefore a deterministic process (except when there exits multiple best choices.) In order to show frequent occurrence of ostracism depending on the initial configuration, Figure 4 gives some exemplary results from a subset of the configuration space. We randomly generate the initial feeling vectors 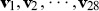 at 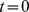 such that their over all liking∶disliking ratio is 3∶7. With those fixed, we vary the initial states of two feeling vectors 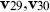. The number of the possible states is 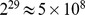. Among these, we pick arbitrary 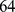 for each and trace the corresponding results. The 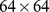 checker board in Figure 4(a) is a visualization of such subset of the configuration space. The each cell represents an initial configuration. If the society with the corresponding initial configuration ends up in one big group, the cell is marked white. The black cells represent initial configurations which lead to a case of ostracism like (c). One can see that if most people are initially biased to unfriendly attitude, ostracism commonly occurs.

It must be noted that even when the target is picked among those late-joiners, it is not necessarily the one who is least similar with others in the beginning: we cannot simply expect who is going to be an outcast from the initial size of the group. If a victim is often accidentally picked, it suggests that ostracism can be understood partly as an inherent property of group formation, not solely as a result of pre-existing social inequalities.

We can also see that, despite its devastating influence, ostracism is originated not directly from negative emotion (hate), but rather from positive one (sense of belonging.) Moreover, this observation is still valid even when people have a guilty of disliking others. Considering that hate is negative emotion that consumes psychological resource, it is natural to modify the payoff function as
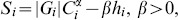
where 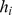 is the number of people that 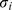 dislikes. Addition of this penalty encourages people to favor others. However, it turned out that all the situations we observed in this section remain essentially the same unless 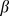 is substantially large. The irony that ostracism can arise even if no one is seeking it appears to have analogies with Schelling's segregation model [20].

### Sacrifice of a Philanthropist

Suppose that there is a person who follows the payoff function (1) with 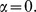 Since the payoff is not affected by congruity, the person is only trying to grow his/her group size. In other words, the person does not mind whether or not the people are similar to him/her and just try to build up mutual friendships with as many people as possible. In this regard, we can call such person with 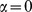 a “philanthropist.”

It is generally accepted that philanthropists who willingly provide their resources with no condition play key roles in social integrity. They help fill the gaps created by market failures and produce social benefits. How to organize philanthropic sectors for a large modern society has become an important issue in public administration and political science.

**Figure 5:**
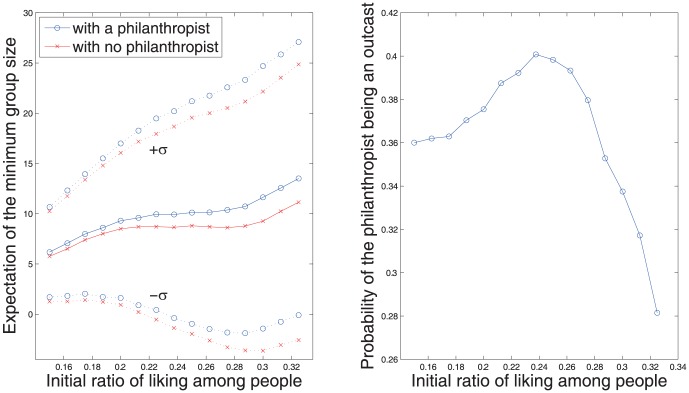
Influence of an philanthropist on the group formation. Except the philanthropist (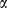 = 0), other people maintain 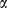 = 1. The group configuration at *t* = 40 is used in the graphs. The results are based on 10,000 realization of the Monte Carlo simulation.

Here we study influence of an single philanthropist on group formation. Let us assume 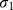 to be a philanthropist with 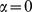 and we set other 29 people 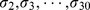 to 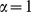 as usual. As mentioned in Section 3, all people tend to conservatively and selectively adjust their social connections when the ratio of liking among people at 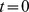 is small (the initial configuration is biased to hate.) Figure 5 depicts some statistics of the resulted group formations according to the initial ratio of friendliness. We are especially interested in how much influence the existence of the philanthropist brings on the minimum size of the groups,
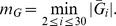
It is impressive that the philanthropist greatly contributes to increase of the minimum group size. See Figure 5(a) for comparison of philanthropy and no philanthropy cases. Two graphs are generated from the Monte Carlo method with 10,000 simulations. When there is a single philanthropist, we generally have larger 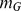. This implies that a philanthropist generally prevents happening of small groups.

Although philanthropy promotes large group formations, it should be noted that the most benefits are taken by the non-philanthropists. That is, philanthropy may have a down side in group formation, especially to whom practices it. Figure 5(b) shows that a philanthropist unavoidably exposes him/herself to a risk of being an outcast from the society. Here the 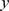 axis stands for the possibility of the sole philanthropist being ended up as an outcast, which reaches as high as 40% for the initial ratio 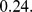 It is somehow irony that individual who pursues for unconditional friendship and help improve overall social integrity risks his/her own social connections.

## Conclusions

In this work, we present a simple model for ostracism formation. It is argued that, as a fundamental desire of human, a sense of belonging is what drives the coalition of the groups and moreover, the occurrence of ostracism. A sense of belonging that an individual experiences consists of two factors, the size and the congruity of the group that he/she belongs to. An attitude toward other people characterizes each individual and enables us to define similarity between individuals. The congruity can be defined as average of similarities with the group members.

Under the synchronized best response rule, people try to maximize their sense of belonging. Sometimes the group size and the congruity are competing and people need to keep balancing between those factors. In order to raise the congruity, they may start to cooperate in rejecting outsiders. This tendency is accelerated as people gather more and more, eventually creating divided groups. When the parameter 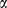 is 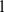 or less, people tend to be generous toward those who are different from them. This generally improves the overall size of the coalitions, but however, still cannot prevent frequent occurrence of an outcast.

Alienating minority is a robust phenomenon in the group formation and occurs even when there is a penalty for disliking others. Since a target could be accidentally chosen with no specific reasons, we can understand ostracism as an inherent part of the group formation. We also study the influence of an philanthropist on the group formation. A philanthropist is defined as a person who pursue for mutual friendships with no condition. Irony of philanthropy is that, while a single philanthropist substantially improves a social integrity, he/she likely faces high risk of being ostracised from the society.
